# Enhancing pharmacogenomic data accessibility and drug safety with large language models: a case study with Llama3.1

**DOI:** 10.3389/ebm.2024.10393

**Published:** 2024-12-03

**Authors:** Dan Li, Leihong Wu, Ying-Chi Lin, Ho-Yin Huang, Ebony Cotton, Qi Liu, Ru Chen, Ruihao Huang, Yifan Zhang, Joshua Xu

**Affiliations:** ^1^ Division of Bioinformatics and Biostatistics, National Center for Toxicological Research, U.S. Food and Drug Administration, Jefferson, AR, United States; ^2^ School of Pharmacy, College of Pharmacy, Kaohsiung Medical University, Kaohsiung, Taiwan; ^3^ Master/Doctoral Degree Program in Toxicology, College of Pharmacy, Kaohsiung Medical University, Kaohsiung, Taiwan; ^4^ Department of Pharmacy, Kaohsiung Medical University Hospital, Kaohsiung Medical University, Kaohsiung, Taiwan; ^5^ Center for Drug Evaluation and Research (CDER), U.S. Food and Drug Administration (FDA), Silver Spring, MD, United States; ^6^ Immediate Office, Office of Translational Sciences, Center for Drug Evaluation and Research, US Food and Drug Administration, Silver Spring, MD, United States

**Keywords:** pharmacogenomics, large language models, LLMs, biomarker, minority ethnic groups

## Abstract

Pharmacogenomics (PGx) holds the promise of personalizing medical treatments based on individual genetic profiles, thereby enhancing drug efficacy and safety. However, the current landscape of PGx research is hindered by fragmented data sources, time-consuming manual data extraction processes, and the need for comprehensive and up-to-date information. This study aims to address these challenges by evaluating the ability of Large Language Models (LLMs), specifically Llama3.1-70B, to automate and improve the accuracy of PGx information extraction from the FDA Table of Pharmacogenomic Biomarkers in Drug Labeling (FDA PGx Biomarker table), which is well-structured with drug names, biomarkers, therapeutic area, and related labeling texts. Our primary goal was to test the feasibility of LLMs in streamlining PGx data extraction, as an alternative to traditional, labor-intensive approaches. Llama3.1-70B achieved 91.4% accuracy in identifying drug-biomarker pairs from single labeling texts and 82% from mixed texts, with over 85% consistency in aligning extracted PGx categories from FDA PGx Biomarker table and relevant scientific abstracts, demonstrating its effectiveness for PGx data extraction. By integrating data from diverse sources, including scientific abstracts, this approach can support pharmacologists, regulatory bodies, and healthcare researchers in updating PGx resources more efficiently, making critical information more accessible for applications in personalized medicine. In addition, this approach shows potential of discovering novel PGx information, particularly of underrepresented minority ethnic groups. This study highlights the ability of LLMs to enhance the efficiency and completeness of PGx research, thus laying a foundation for advancements in personalized medicine by ensuring that drug therapies are tailored to the genetic profiles of diverse populations.

## Impact statement

This study demonstrates the utility of Large Language Models (LLMs), specifically Llama3.1-70B, in automating pharmacogenomic (PGx) data extraction, addressing the limitations of traditional manual methods that are labor-intensive and slow to update. By achieving high accuracy in identifying drug-biomarker pairs and integrating diverse data sources, this work offers a practical solution for pharmacologists, regulatory agencies, and healthcare professionals to streamline PGx database updates. With automated extraction processes, LLMs reduce the time and effort required to incorporate new PGx insights, potentially enabling updates at a frequency and scale that were previously unfeasible. This capability is critical for translating PGx research into actionable, personalized treatment guidelines that reflect the genetic diversity of patient populations, ultimately advancing equity in personalized medicine.

## Introduction

Pharmacogenomics (PGx) represents a pivotal advancement in personalized medicine, tailoring drug therapies based on an individual’s genetic profile [1, 2]. By understanding how genetic variations influence drug response, PGx enables healthcare providers to optimize treatment efficacy and minimize adverse drug reactions [3, 4]. This personalized approach holds the potential to significantly enhance patient outcomes, especially in the management of complex diseases such as cancer, cardiovascular disorders, and mental health conditions [5]. The importance of PGx lies in its ability to provide more precise and effective treatments. For instance, variations in genes encoding drug-metabolizing enzymes, drug transporters, and drug targets can greatly influence a patient’s response to certain medications. These genetic differences can determine whether a patient will benefit from a particular drug, experience no effect, or suffer from adverse reactions [6, 7].

Despite its promise, the clinical implementation of PGx has been slower than anticipated, partly due to the complexity of drug-gene interactions and the need for extensive empirical evidence [8]. As our understanding of genetic factors in drug response continues to grow, PGx is poised to become a standard component of healthcare, revolutionizing the way treatments are tailored to individual patients. Various databases and resources for PGx information have been established to improve the accessibility and utility of this data. Key resources include the Pharmacogenomics Knowledgebase (PharmGKB), which curates information about how genetic variations affect drug response [9]. The pharmacogenomics database (PGxDB) database offers a comprehensive platform for integrating PGx data, allowing researchers to explore drug, target, and disease relationships [10]. Additionally, the FDA has released the Table of Pharmacogenomic Biomarkers in Drug Labeling (Table of Pharmacogenomic Biomarkers in Drug Labeling | FDA), providing drug and PGx biomarker pairs found in given drug labeling sections which serves as the primary data source for this study. Meanwhile, PGx-related research articles containing new findings and conclusions are crucial for timely updating of current PGx information. For instance, relevant abstracts can be retrieved from PubMed or other resources. These resources are essential for advancing the field of PGx and ensuring that clinicians have the necessary tools to apply genetic insights to patient care.

Large Language Models (LLMs) like Llama3.1 represent a significant advancement in natural language processing, offering powerful capabilities for extracting and analyzing complex data from diverse sources. These models, trained on vast amounts of text, can understand and generate human-like language, making them highly effective for tasks such as data extraction, summarization, and information synthesis [11, 12]. Recent studies have demonstrated the potential of LLMs in various fields, including PGx. For instance, LLMs have been shown to significantly improve the efficiency and accuracy of data extraction processes, and AI assistant showed improved efficacy in answering user questions [13]. By leveraging these models, researchers can automate the extraction of PGx information, overcoming challenges related to the time-consuming and labor-intensive nature of manual data processing.

In this study, we focused on evaluating the capabilities of LLMs, particularly Llama3.1-70B [14, 15], for PGx information extraction from various data sources. Our goal was to enhance the current PGx information collection by improving its accuracy and incorporating recent studies to fill in gaps and ensure the data is comprehensive. It was essential to ensure that the model could reliably identify and extract key PGx data, such as drugs and related biomarkers, from diverse sources with a remarkable degree of precision. The model demonstrated a high accuracy rate of 91.4% when extracting information from structured texts in the FDA PGx Biomarker table and 82% from the mixed texts, underscoring its effectiveness in handling different types of data.

A key aspect of our study was the integration of diverse resources, including well-structured databases like the FDA PGx Biomarker table, alongside relevant scientific abstracts. By combining these sources, we were able to cross-validate and enrich the PGx data, ensuring a more comprehensive, accurate, and up-to-date dataset, particularly with insights related to underrepresented populations and novel drug-biomarker interactions. The results can better support personalized medicine initiatives and enhance the overall effectiveness of pharmacogenomic applications.

## Materials and methods

### Data processing for the FDA PGx biomarker table

The FDA PGx Biomarker table (06/2023 version) was downloaded in PDF format and converted into one Excel table. All the special characters were then removed from the texts. Biomarkers with multiple gene names or aliases were further processed to ensure all the entries were retained. For instance, for the listed biomarker ERBB2 (HER2), either ERBB2 or HER2 identified by the model was considered a correct identification. To ensure there was sufficient content from which the model could extract information, labeling texts in the FDA PGx Biomarker table with fewer than 300 words were removed from the analysis.

### Prompt and model settings

The Llama3.1-70B-Instruct model [14, 15] was employed in this study for the PGx information extraction and summarization. The model was run using its default settings. We utilized the “client.chat.completions.create” function to interact with the model and obtain the responses. To guide the model effectively, we set the system context as: “You are an expert in pharmacogenetics and assist me in extracting information from texts.” This context was designed to align the model’s responses with the specialized nature of the task. The PGx texts from the PGx Biomarker table that required information extraction, along with specific questions, were provided in the prompt as user content. For example, a typical prompt would be: “*Please review this labeling text and identify the pairs of drug and biomarker clearly mentioned. Output the pairs in ‘drug-biomarker’ format. Please try to give me both the generic name and brand name of the drug.*” As a result, the model may identify multiple drug-biomarker pairs from the query texts, and we consider the extraction correct if the listed pair is included in the results.

The prompt we used to extract PGx information from the label texts was “*Based on this content [texts for information extraction], answer the following questions step-by-step in short answers, only about the drug [drug name] and biomarker [biomarker name] as a pair. Then please generate a horizontal form table with the following items: Phenotypes/Genotypes: Identify the phenotypes (drug response influenced) or genotypes (genetic variants) associated with the biomarker. Frequency by Ethnicity: Provide the frequencies of the identified phenotypes or genotypes by ethnicity. Reason for PGx Labeling: State the reason for pharmacogenomic labeling of the biomarker. ADRs Associated with Biomarker: Identify adverse drug reactions related to the biomarker. Gender Differences: Indicate whether the biomarker is influenced by gender (Yes/No). Ethnicity Differences: Indicate whether drug response differs by ethnicity (Yes/No). Asian Stats: Provide the phenotype or genotype frequency of the biomarker in the Asian population. If no data is available, write ‘No data.’ Black/AA Stats: Provide the phenotype or genotype frequency of the biomarker in the Black population. If no data is available, write ‘No data.’ Hispanic Stats: Provide the phenotype or genotype frequency of the biomarker in the Hispanic population. If no data is available, write ‘No data.’ Polymorphism: Identify the genotype of the biomarker that influences drug response. Summary: Categorize the information using one or more keywords from ‘Therapeutic Use,’ ‘Dosing,’ ‘Drug Response,’ ‘Metabolism,’ and ‘Ethnicity-Specific’.*”

### Generation of mixed texts

To mimic the real-world scientific texts, which often discuss multiple drugs and biomarkers, we generated mixed texts by combining the labeling texts associated with two different drug-biomarker pairs from the FDA PGx Biomarker table. Each labeling text record was divided into five groups by randomly determining where to break the text, always ensuring the breaks occurred at the end of a sentence. This approach preserved the original sequence of sentences within each group. To create a mixed text, we selected these ten groups, five from each of two different segmented records, and merged them. This process allowed us to generate new, coherent mixed texts while blending information from two distinct drug-biomarker pairs (Supplementary Figure 1).

### PubMed abstracts query

The PubMed API and Entrez library [16] were used to retrieve relevant abstracts based on a given drug-biomarker pair. We requested the title or abstract of one publication to contain both the drug and biomarker. To further narrow down the candidates to ensure the relevance of the collected abstracts, we also required that one of the keyworks, including PGx, pharmacogenomics, minority, variants, mutations, and population, be presented in either the title or abstract. Additionally, if no abstract could be found based on the initial query, we then searched for those abstracts that mentioned only the drug and biomarker. Considering the limitation of prompt length of the Llama3.1-70B model, we collected up to five abstracts for each PGx labeling record. The same prompt was used to extract PGx information from abstracts and from labeling texts.

### Calculation of concordance rate

In this study, we used the concordance rate to measure the extent to which PGx categories (Therapeutic Use, Dosing, Drug Response, Metabolism, and Ethnicity-Specific) identified from the PGx labeling texts were also represented in the relevant abstracts for the same drug-biomarker pair. The concordance rate was calculated using the following formula:

$$Concordance\;rate=\frac{\#\text{of}\;\text{PGx}\;\text{categories}\;\text{common}\;\text{to}\;\text{both}\;\text{PGx}\;\text{labeling}\;\text{texts}\;\text{and}\;\text{relevant}\;\text{abstracts}}{\#\text{of}\;\text{PGx}\;\text{categories}\;\text{identified}\;\text{in}\;\text{PGx}\;\text{labeling}\;\text{texts}}$$

This metric provided a clear and quantitative assessment of the overlap between the information in the PGx labeling texts and the scientific abstracts, allowing us to evaluate the consistency and completeness of the extracted data across different sources.

## Results

### High accuracy achieved with structured labeling texts in the FDA PGx biomarker table

**FIGURE 1:**
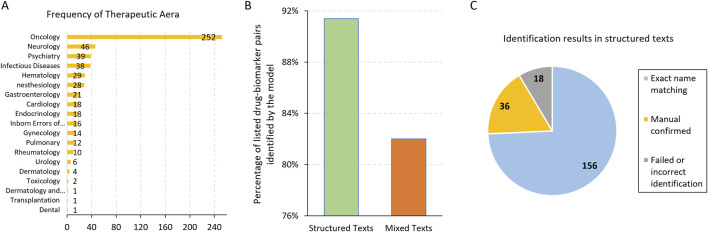
**(A)** The frequency of the Therapeutic Area in the FDA PGx Biomarker table. Majority of the records were related to Oncology. **(B)** The percentage of listed drug-biomarker pairs identified correctly by the model in structured and mixed texts, respectively. **(C)** The number and partition of the drug-biomarker identification results in structured texts.

We first evaluated the model’s ability to identify drug and biomarker pairs from the labelling texts in the FDA PGx Biomarker table. Each entry contains the drug name, associated biomarker, therapeutic area, and labeling texts. Our analysis focused on the therapeutic area of Oncology, which had the largest number of records in the table (Figure 1A). We excluded records with non-gene biomarkers such as chromosome alterations or hormone receptors. As a result, out of 210 drug-biomarker pairs, the model successfully identified 192 pairs, achieving an identification accuracy of 91.4% (Figure 1B). Among these, 36 pairs required manual review and confirmation due to discrepancies arising from variations in nomenclature, such as the use of generic versus brand names of drugs or biomarker aliases. For example, the model identified the biomarker MKI67 as Ki-67, where MKI67 refers to the gene encoding the Ki-67 protein, indicating both terms represent the same entity. After manual validation, these 36 pairs missed by exact name matching were confirmed as correctly identified, contributing to the overall count of 192 accurate predictions (Figure 1C).

By manually reviewing the 18 records where the model failed to identify the drug-biomarker pairs, we found that most of them had short labeling texts in the FDA PGx Biomarker table, sometimes without the drug or biomarker even mentioned, leaving no way for the model to extract them. Another example was the drug brand name LONSURF, which was mentioned in the labeling text column of the PGx Biomarker table, but the listed drug names were tipiracil and trifluridine, the generic names of this drug. For this particular record, the model failed to identify either the brand or generic names.

### Challenges with mixed texts

As Llama3.1-70B demonstrated high accuracy in identifying drug-biomarker pairs from a section of labeling text, we further challenged the model with mixed texts from two records. This approach aimed to mimic the complex content often encountered in scientific studies, where discussions typically involve multiple drugs and biomarkers. To create a mixture testing set, we selected two records, each related to different drugs, and split them by sentences. These sentences were then merged to form a single paragraph, which was subsequently fed to the model (Methods, Supplementary Figure 1). This setup was designed to evaluate the model’s ability to accurately extract relevant drug-biomarker pairs from a less structured and more intricate text, closely resembling real-world scientific documentation.

From the 156 records where the model correctly identified the drug-biomarker pairs without manual confirmation, we generated 50 mixture texts for testing (Methods). Using the same prompt and manual validation, we observed that the model could accurately identify at least one drug-biomarker pair for the testing records in 41 out of 50 (82%) cases (Figure 1B). Specifically, the model identified all the two drug-biomarker pairs in 32 records (64%), indicating a relatively high level of accuracy even with mixed and more complex text inputs. However, some cases posed significant challenges for the model. For instance, fusion names like BCR-ABL1 were occasionally difficult for the model to identify correctly. Additionally, there were instances where the model misidentified drugs due to the complexity of the text. In one particular case, a record included two drugs: ALIMTA (the brand name for pemetrexed) and pembrolizumab, which was mentioned as a comparator drug in the study. The primary drug for this record was pemetrexed, but the model incorrectly identified pembrolizumab as the paired drug. Notably, the drug-biomarker pair for this challenging case had been correctly identified in previous assessments without the interference of another record.

We further evaluated the mis-identified drug-biomarker pairs in the mixture texts by examining cases where the model incorrectly linked the drug and biomarker from two different records. As a result, ten mis-linked drug-biomarker pairs were identified from nine records. The results suggest that the presence of unrelated content may confuse the model, highlighting the need for careful consideration when handling complex and mixed information in texts.

### Extraction of PGx information related to minority groups

Pharmacogenomics information is crucial for understanding how genetic variations influence drug responses across different population groups. Many PGx studies highlight the role that ethnic differences may play in drug efficacy and safety, with some of these findings reflected in labeling documents. Unique genetic profiles that may significantly impact responses to medications have been observed among minority groups, though these profiles remain underexplored. Despite growing awareness of genetic diversity, many minority populations continue to be underrepresented in PGx research, contributing to gaps in personalized medicine.

In this study, we collected 178 records from the FDA PGx Biomarker table containing terms such as “American,” “Asian,” “Caucasian.” For each labeling text, we tasked the model to extract PGx information related to race or ethnicity. Key information extracted included the presence of ethnicity differences, frequency of genetic variants by ethnicity, reasons for PGx labeling, and adverse drug reactions (ADRs) associated with biomarkers (as detailed in Table 1). The model demonstrated its effectiveness by accurately identifying crucial details, such as the phenotypes of Poor Metabolizers (PM) and Extensive Metabolizers (EM) for the tolterodine-CYP2D6 pair. It correctly highlighted that the tolterodine labeling indicates approximately 7% of Caucasians and 2% of African Americans were poor metabolizers in that study. It is important to acknowledge that this labeling uses outdated terminology. The terms “White” and “Black/African American” are now preferred. This differentiation is vital for understanding the potential risks of adverse reactions, like QT prolongation, in specific populations (Table 1).

**TABLE 1:** An example of the PGx information extracted from the FDA PGx Biomarker table related to the give drug-biomarker pair of Tolterodine-CYP2D6.

Pair	Tolterodine-CYP2D6
Phenotypes/Genotypes	Poor metabolizers (PM), Extensive metabolizers (EM)
Frequency by Ethnicity	Approximately 7% of Caucasians, approximately 2% of African Americans
Reason for PGx Labeling	Increased risk of QT prolongation and higher serum concentrations of tolterodine in poor metabolizers
ADRs Associated with Biomarker	QT prolongation, increased risk of cardiac arrhythmias
Gender Differences	No
Ethnicity Differences	Yes
Asian Stats	No data
Black/AA Stats	Approximately 2%
Hispanic Stats	No data
Polymorphism	CYP2D6 poor metabolizers have a slower rate of tolterodine metabolism, resulting in higher serum concentrations
Summary	Metabolism, Dosing, Drug Response, Ethnicity-Specific

We assessed the model’s accuracy in determining whether there were “ethnicity differences” in the labeling text column. The model was asked to answer a Yes/No question (Table 1) based on whether any information on ethnicity difference was found in the texts (Methods). Of the 178 records analyzed, 94 contained information explicitly stating ethnicity differences. However, some records mentioned the inclusion of diverse minority groups in studies but did not discuss or conclude any differences among these groups. For example, a labeling might state “*56 of the subjects were male, 61 were White, 20 were Black or African American, 8 were Hispanic or Latino*” but if no comparisons or outcomes were discussed, it should be marked as having no ethnicity difference.

We then manually reviewed the records classified by the model as having no ethnicity difference, identifying any false negatives. Impressively, the model achieved 100% accuracy in correctly identifying records that explicitly stated ethnicity differences. This finding underscores the model’s reliability in detecting ethnicity-related PGx information and highlights the importance of ensuring accurate representation and consideration of minority groups in PGx research. This work illustrates the value of using LLMs to systematically and accurately identify PGx information across diverse populations. With appropriate data, LLMs have the potential to retrieve important PGx insights for minority groups from diverse published sources, contributing to more inclusive and equitable healthcare practices.

### Validation of extracted PGx information

The extracted data, encompassing details about drug-biomarker pairs, genetic variations, and ethnicity-specific information, plays a vital role in personalized medicine, which requires high accuracy. While verifying straightforward elements identified by the model, such as the presence or absence of ethnicity differences, is relatively easy, evaluating the detailed PGx information extracted from the texts is challenging due to its complexity. The intricacies involved in interpreting genetic data and its clinical implications require careful consideration. Manually verifying the extracted information would be impractical given the large volume and complexity of the data. Therefore, we implemented a systematic validation process using predefined PGx categories to evaluate the accuracy and consistency of the extracted information. This approach ensured a thorough and efficient assessment, allowing us to confirm the reliability of the model’s outputs.

Particularly, when we tasked the model with extracting PGx information from the labeling texts in the FDA PGx Biomarker table, we also required a summary of each record using predefined keywords, including Therapeutic Use, Dosing, Drug Response, Metabolism, and Ethnicity-Specific (Table 1). For each ethnic PGx record, we collected up to five PubMed abstracts that contained the drug-biomarker pair in the title or abstract. To address concerns that abstracts might focus on different aspects and to narrow down the search to more relevant studies, we included additional keywords such as pharmacogenomics, PGx, and minority, in the PubMed query (Methods). This approach increased the chances of retrieving abstracts that provided the necessary PGx details, ensuring a thorough and focused validation process.

As a result, 137 out of 178 ethnic records had at least one abstract found in PubMed that contained the drug-biomarker pairs. The Llama3.1-70B model was then tasked again to tag each individual abstract with the predefined PGx information categories. By comparing the categories from the FDA PGx Biomarker table with those from the relevant abstracts, we evaluated the accuracy and consistency of the extracted information, ensuring alignment with external authoritative sources. A matched PGx category indicates that the particular drug-biomarker pair was studied by different research groups and that similar findings were concluded in the PGx field.

**FIGURE 2:**
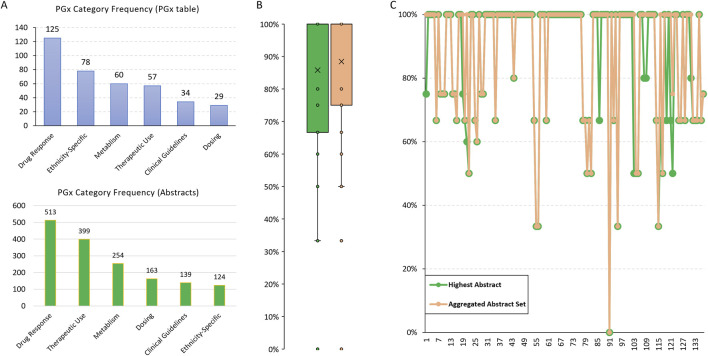
PGx categories summarized from the FDA PGx Biomarker table and relevant scientific abstracts. **(A)** The frequency of predefined PGx categories summarized by Llama3.1-70B for the 178 ethnic records from the FDA PGx Biomarker table. **(B)** The concordance rate of PGx categories between the FDA PGx Biomarker table and abstracts. The highest rate based on a single abstract and the rate based on an aggregated abstract set were compared. **(C)** A comparison of the highest and the aggregated concordance rate for each individual record.

Among the 178 ethnic records in the FDA PGx Biomarker table, 125 discussed Drug Response, making it the most frequently mentioned category (Figure 2A). Additionally, we found a high consistency in that 78 out of 94 records (83%) identified with Ethnicity Differences were categorized as Ethnicity-Specific. In contrast, only 29 records were related to Dosing. However, the abstracts we collected, which involved the same drugs and biomarkers, exhibited different frequency patterns for these PGx categories (Figure 2A). The lower frequency of ethnicity-specific data in the abstracts suggests that this aspect may not be a major focus in the studies we collected.

We then calculated the PGx categories concordance rate, defined as the percentage of the categories identified in PGx labeling that were also covered by those from relevant abstracts. To assess the consistency of the extracted information, we compared the highest concordance rate based on a single abstract and the rate based on the aggregated abstract set. The median consistency was over 85% (Figure 2B), indicating high accuracy of the PGx information extracted by the LLM. This cross-validation not only confirms the reliability of the model’s extraction capabilities but also highlights the robustness of our methodology in integrating and validating pharmacogenomic data across diverse sources.

No big difference was observed between the highest and aggregated concordance rates (Figure 2C), suggesting that individual abstracts are sufficiently comprehensive in covering the relevant PGx categories. The approach we used successfully retrieved abstracts that were well-aligned with the information we were interested in from the FDA PGx Biomarker table, ensuring that the abstracts are relevant and valuable for validating the PGx information.

The findings indicate that we can use these abstracts complementarily with the labeling texts to potentially extract additional PGx information for certain drug-biomarker pairs. As shown in Table 2, we asked the model the same questions based on the integrated texts of the four relevant abstracts (PMIDs: 28087463, 24619889, 22277677, 14606931). Additional PGx information associated with ethnic groups of Japanese and Chinese were found in these abstracts.

**TABLE 2:** An example of the PGx information extracted from the FDA PGx Biomarker table and relevant abstracts collected from PubMed for Tolterodine-CYP2D6.

Pair	Tolterodine-CYP2D6
Phenotypes/Genotypes	Extensive metabolizers (EM), Poor metabolizers (PM), Intermediate metabolizers (IM), Ultra-rapid metabolizers (UM), Variants: CYP2D6*2, CYP2D6*10, CYP2D6*92, CYP2D6*93, CYP2D6*94, CYP2D6*95, CYP2D6*96, F164L, F219S, D336N, E215K
Frequency by Ethnicity	CYP2D6 poor metabolizers: 5-10% in Caucasians, 1-2% in Asians, No data for other ethnicities
Reason for PGx Labeling	Variability in metabolism leading to differences in drug response and adverse reactions
ADRs Associated with Biomarker	Increased risk of adverse reactions in PMs due to higher plasma concentrations. Decreased efficacy in UMs due to lower plasma concentrations
Gender Differences	No
Ethnicity Differences	Yes, CYP2D6 allele frequencies vary among ethnicities
Asian Stats	CYP2D6*10: 51.4% (Japanese), 40.9% (Chinese) * CYP2D6 poor metabolizers: 1-2%
Black/AA Stats	No data
Hispanic Stats	No data
Polymorphism	Variants: CYP2D6*2, CYP2D6*10, CYP2D6*92, CYP2D6*93, CYP2D6*94, CYP2D6*95, CYP2D6*96, F164L, F219S, D336N, E215K
Summary	Metabolism, Drug Response, Ethnicity-Specific

## Discussions

While it is relatively straightforward to validate the extraction of certain PGx items from structured texts, such as drug and biomarker names from labeling sections, assessing the overall quality and completeness of the extracted information from more variable sources poses significant challenges. Unlike structured data, where predefined formats facilitate comparison and validation, publications and reports vary widely in focus and detail, complicating direct comparison of PGx information across different sources. To address this challenge, we employed a strategy where the model was instructed to tag the extracted texts with predefined categories, enabling a more systematic comparison. This tagging approach offers an initial method for aligning information across sources; however, we recognize that these categories may require further refinement or customization based on the specific content and objectives of different studies. Our results demonstrated that Llama3.1-70B achieved high accuracy in extracting drug and biomarker pairs from structured labeling texts, particularly when biomarkers were listed as gene or protein names. However, the model encountered difficulties when extracting less common biomarker names, such as “hormone receptors,” which were excluded from the main analysis due to lower extraction accuracy. This limitation highlights the importance of prompt engineering and model tuning for specific use cases. Tailoring prompts to explicitly account for uncommon biomarkers or providing additional context within the prompt could improve the model’s ability to accurately identify and extract these entities, an approach that warrants further exploration.

Identifying drug-biomarker pairs in mixed texts, where multiple records are combined, presents a more complex challenge for LLMs. Our study found that while Llama3.1-70B performed well with structured labeling texts, its accuracy decreased when processing mixed texts, likely due to the increased ambiguity and variety of content. This challenge would likely increase further with full-text publications, where drug-biomarker relationships are not always clearly delineated. To address these complexities, future studies could be benefit from a targeted approach, such as instructing the model to focus on specific drug-biomarker pairs to enhance extraction accuracy. In preliminary tests, the model was able to accurately identify relevant information from mixed texts when a specific drug-biomarker pair was targeted, suggesting that targeted prompts could improve accuracy in more complex texts.

Our findings demonstrate that LLMs like Llama3.1-70B can efficiently support the extraction of PGx information from structured sources, such as the FDA PGx Biomarker table, providing a foundation for integrating valuable data from scientific abstracts and potentially, with further refinement, from more complex sources like full-text publications. This automated approach can reduce the time and effort required for initial data extraction, improving the completeness of PGx databases by streamlining the process. However, we recognize that integrating LLM-extracted data directly into regulatory or clinical decision-making frameworks would require extensive validation and quality control, including human oversight, to ensure accuracy and relevance.

Implementing a structured workflow that leverages LLMs for routine extraction of PGx data could support the initial stages of database updates. Such a process would involve combining LLM-extracted insights with manual review and verification steps, enhancing the accessibility and usability of PGx data for non-regulatory applications, such as research and exploratory analyses in pharmacogenomics. This framework can be refined to incorporate more sophisticated validation methods, advancing the field of personalized medicine incrementally through a combination of automated and manual processes. Future work will focus on evaluating and refining this workflow to ensure reliability and utility in various PGx contexts.

While our study utilizes the Llama3.1-70B model, the primary focus of this work is the development of a generalizable framework for pharmacogenomic (PGx) data extraction. Our approach, which involves structured prompts, data integration techniques, and strategies for handling complex, mixed-text data, is designed to be adaptable to future advancements in LLM technology. As LLMs continue to improve, this framework can be applied to newer models, enabling consistent, automated PGx data extraction and updating without reliance on a specific LLM version. This flexibility makes the framework suitable for various applications in PGx research, supporting the evolving needs of pharmacologists, regulatory bodies, and healthcare researchers.

## Data Availability

The original contributions presented in the study are included in the article/Supplementary Material, further inquiries can be directed to the corresponding authors.
