# Supplementary figures and images for: Enhancing pharmacogenomic data accessibility and drug safety with large language models: a case study with Llama3.1
Source: Exp Biol Med (Maywood). 2024 Dec 3;249:10393. doi: 10.3389/ebm.2024.10393 (PMC11650518; doi:10.3389/ebm.2024.10393)

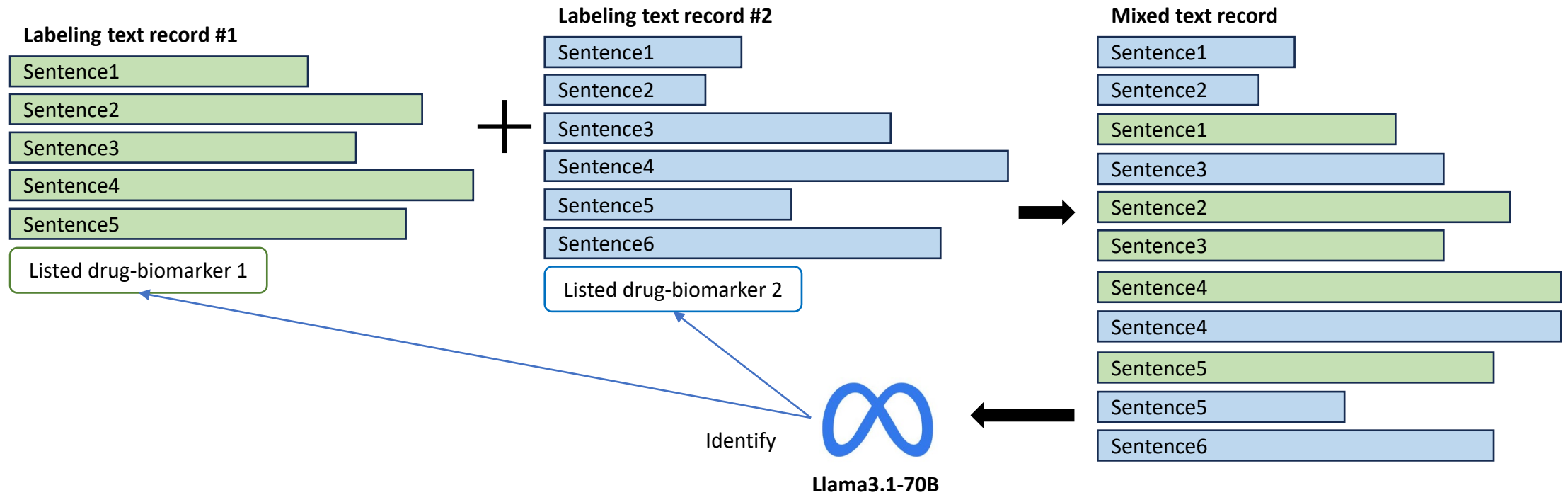

**Supp. Figure1.** An illustration of the process of merging two labeling text records.

Supplement: Supplementary file 1 [file DataSheet1.PDF]
